# Ferulic Acid Combined With Bone Marrow Mesenchymal Stem Cells Attenuates the Activation of Hepatic Stellate Cells and Alleviates Liver Fibrosis

**DOI:** 10.3389/fphar.2022.863797

**Published:** 2022-05-19

**Authors:** Rui Zhang, Wenhang Li, Xiaodan Jiang, Xinyi Cui, Hongjie You, Zuoqing Tang, Wenlan Liu

**Affiliations:** ^1^ School of Traditional Chinese Medicine, Capital Medical University, Beijing, China; ^2^ School of Basic Medical Sciences, Capital Medical University, Beijing, China

**Keywords:** liver fibrosis, BMSCs, ferulic acid, HSCs, miR-19b-3p

## Abstract

Bone marrow mesenchymal stem cells (BMSCs) can effectively alleviate liver fibrosis, but the efficacy of cell therapy alone is insufficient. In recent years, a combination of traditional Chinese medicine (TCM) and cell therapy has been increasingly used to treat diseases in clinical trials. Ferulic acid (FA) is highly effective in treating liver fibrosis, and a combination of cells and drugs is being tested in clinical trials. Therefore, we combined BMSCs and Ferulic acid to treat CCl_4_-induced fibrosis and determine whether this combination was more effective than single treatment. We used BMSCs and FA to treat CCl_4_-induced fibrosis in rat models, observed their therapeutic effects, and investigated the specific mechanism of this combination therapy in liver fibrosis. We created a BMSC/hepatic stellate cell (HSC) coculture system and used FA to treat activated HSCs to verify the specific mechanism. Then, we used cytochalasin D and angiotensin II to investigate whether BMSCs and FA inactivate HSCs through cytoskeletal rearrangement. MiR-19b-3p was enriched in BMSCs and targeted TGF-β receptor II (TGF-βR2). We separately transfected miR-19b-3p into HSCs and BMSCs and detected hepatic stellate cell activation. We found that the expression of the profibrotic markers α-SMA and COL1-A1 was significantly decreased in the combination group of rats. α-SMA and COL1-A1 levels were also significantly decreased in the HSCs with the combination treatment. Cytoskeletal rearrangement of HSCs was inhibited in the combination group, and RhoA/ROCK pathway gene expression was decreased. Following angiotensin II treatment, COL1-A1 and α-SMA expression increased, while with cytochalasin D treatment, profibrotic gene expression decreased in HSCs. The expression of COL1-A1, α-SMA and RhoA/ROCK pathway genes was decreased in the activated HSCs treated with a miR-19b-3p mimic, indicating that miR-19b-3p inactivated HSCs by suppressing RhoA/ROCK signalling. In contrast, profibrotic gene expression was significantly decreased in the BMSCs treated with the miR-19b-3p mimic and FA or a miR-19b-3p inhibitor and FA compared with the BMSCs treated with the miR-19b-3p mimic alone. In conclusion, the combination therapy had better effects than FA or BMSCs alone. BMSC and FA treatment attenuated HSC activation and liver fibrosis by inhibiting cytoskeletal rearrangement and delivering miR-19b-3p to activated HSCs, inactivating RhoA/ROCK signalling. FA-based combination therapy showed better inhibitory effects on HSC activation.

## Introduction

Hepatic fibrosis is a repair response of the liver to chronic hepatocyte injury. Following persistent injury of the liver or stimulation by fibrotic factors, pathological changes in the extracellular matrix (ECM) occur in the liver tissue, and the balance between the production or degradation of ECM is disrupted, leading to excessive deposition of ECM in the liver tissue, fibrosis and eventually cirrhosis. (Bataller and Brenner 2005; M. Pinzani and Rombouts, 2004). Activated hepatic stellate cells (HSCs) are an important cause of liver fibrosis. Upon liver injury, Kupffer cells and endothelial cells in the liver release cytokines, such as transforming growth factor-β (TGF-β), to activate quiescent HSCs. Activated HSCs transdifferentiate into myofibroblastic HSCs (Virarkar et al., 2021). Then, the cytoskeleton in these HSCs undergoes rearrangement, the cell dynamics increase, and these cells migrate to the site of liver injury and produce substantial amounts of ECM components, such as α-smooth muscle actin (α-SMA) and collagens. Fired-Man proved that activated HSCs are the main source of extracellular collagen in the liver. Therefore, regulating HSC activation and migration could be an anticipated antifibrotic strategy.

Bone marrow mesenchymal stem cells (BMSCs) have become an emerging therapeutic agent for liver fibrosis in recent years due to their proliferative potential, low immunogenicity, abundant sources, tissue repair ability, and chemotactic and homing abilities (Alfaifi et al., 2018). The therapeutic effects of MSCs on liver fibrosis are related to their ability to undergo hepatocyte-like differentiation, participate in immunization activities and secrete paracrine factors (Yao et al., 2020). For example, hBMSC-derived exosomes protected against liver fibrosis by regulating the Wnt/β-catenin pathway, and antler stem cells were identified as a novel source to reduce liver fibrosis (Rong et al., 2019; Rong et al., 2020). In our previous study, BMSCs ameliorated CCl_4_-induced liver damage and fibrosis in rats *via* the FGF2-Dlk1-Notch1 pathway (Li et al., 2017).

Although BMSCs can improve liver fibrosis, one of the major challenges of BMSCS therapy is the inefficiency of clinical treatment. BMSCs are commonly injected at 1 × 10^6^ cells/kg. BMSC treatment can also lead to potential risks, such as *in vitro* cell amplification and adverse reactions caused by implanted BMSCs. In addition, several phase III clinical trials have failed due to the inefficiency of BMSCs. Therefore, identification of strategies to improve the therapeutic effects has become a key issue in BMSC therapy (Ankrum and Karp 2010; Liao et al., 2017). In recent studies, traditional Chinese medicine (TCM) combined with BMSC therapy has shown unique advantages and characteristics. Bie jia jian pill combined with BMSCs suppressed hepatocellular carcinoma (Jingjing et al., 2021). Saponins from *Rhizoma Panacis Majoris* combined with BMSCs could improve hepatic fibrosis (Tian 2016). Naringin combined with BMSCs and acellular dermal matrix affected cartilage injury (Ye Chao et al., 2021). Ferulic acid (FA), as an important ingredient in TCM, has therapeutic activity against a variety of diseases. The hepatoprotective effects of FA have been demonstrated in studies using CCl_4_ to induce liver fibrosis (Jayamani et al., 2018; Ali et al., 2021; Li et al., 2021). Therefore, we proposed the combination of BMSCs and FA in the treatment of liver fibrosis and investigated the mechanisms underlying the antifibrotic effect in this study.

## Materials and Methods

### Cell Culture

SD-BMSCs were isolated from the bone marrow of Sprague-Dawley (SD) rats aged 3–6 weeks and were cultured as previously described (Yuhui Lin et al., 2011). BMSCs were cultured in Dulbecco’s modified Eagle’s medium (DMEM; Gibco, MA, United States, CAT: C11995500BT) with 10% foetal bovine serum (FBS; AQ, Beijing, China, CAT: AQmv02200-500ml) and 1% penicillin/streptomycin (P/S; Gibco, CAT: 15140122) at 37°C in an incubator containing 5% CO_2_. HSC-T6, a rat HSC cell line (purchased from Keygen Biotech, Nanjing, China, CAT: KG313), was cultured in DMEM-high (Gibco) with 10% FBS (AQ) and 1% P/S (Gibco) at 37°C in a humidified atmosphere containing 5% CO_2_. SD-BMSCs and HSC-T6 cells were seeded in 25-cm^2^ plates at 70% confluence.

### Experimental Animal Models

Male SD rats were treated in the Experimental Animal Center of Capital Medical University in an environment without a specific pathogen barrier. All experiments were approved and supervised by the Animal Care and Use Committee of Capital Medical University (approval number: SCXK 2016-0006), and the animals were housed in a facility with a 12 h light/12 h dark cycle and allowed free access to food and water. Eight-week-old rats (*n* = 30) received 0.2 ml/kg body weight of CCl_4_ (RhawnSeal, Shanghai, China, CAT: R015578-500ml) dissolved in olive oil by intraperitoneal injection for 2 weeks, followed by 0.1 ml/kg body weight of CCl_4_ for 2 weeks and finally 0.05 ml/kg body weight of CCl_4_ for 2 weeks. As a normal control (NC group), rats were injected with an equal volume of olive oil (*n* = 6). All modelling injections were administered three times per week for a total of 6 weeks. After 6 weeks of modelling, 30 model rats were randomly divided into 5 groups (*n* = 6). We randomly chose rats in 2 CCl_4_ groups (*n* = 6) by injection of 5 × 10^6^ BMSCs into tail vein (stained with CFDA SE, Meilunbio, Dalian, China, CAT: MB2308-1) once a week for 2 weeks. Then, we established a CCl_4_ group and a CCl_4_+ BMSC group (LIU Wen-lan et al., 2014), and the animals were treated with FA (10 mg/kg) by intragastric administration (Solarbio, Beijing, China, CAT: F8330-5g). The rats in another CCl_4_ group were treated with colchicine (0.1 mg/kg) (Aladdin, Shanghai, China, CAT: C106738-100mg) as a positive control (Mu et al., 2018). The animals in the other groups were treated with equal amounts of normal saline. All rats were gavaged once a day for 2 weeks. After treatment for 2 weeks, all rats were sacrificed, and the serum and liver tissues were obtained. The groups were as follows: normal control group (NC), model group (model), colchicine group (positive control), FA group (FA), BMSC group (BMSCs), and BMSC + FA group (FC).

### Measurement of Aspartate Aminotransferase and Alanine Aminotransferase

We collected 1 ml of blood from each animal. After storage at room temperature for 4 h, the serum was separated by centrifugation (×1500 g, 10 min). Then, 200 μL serum samples were used for analysis. The activities of serum aspartate aminotransferase (AST) and alanine aminotransferase (ALT) were determined by commercial kits obtained from Konkahongyuan Co. (Beijing, China, CAT: KA4189) according to the manufacturer’s instructions.

### Histopathological Evaluation

Fresh liver tissue samples were fixed in 10% formaldehyde at 4°C overnight and embedded in paraffin wax for histological evaluation. Sections were stained with haematoxylin and eosin (HE), and the severity of the histological changes was evaluated. We obtained images by using a microscope equipped with a LI-COR Odyssey Infrared Imaging System (LI-COR, Inc., Lincoln, NE, United States).

### Masson Trichrome Staining

For staining, 4–6 µm-thick sections were obtained from paraffin-embedded blocks and stained with Masson trichrome (MT) and FANCM. For MT staining, the Trichrome III Green Staining Kit (Solarbio, Beijing, China, CAT: G1340), a modified version of the MT stain, was used with Bouin’s solution to intensify the final colouration.

### Cell Coculture Experiments

HSCs were activated with TGF-β1 (10 ng/ml, PeproTech, United States, CAT: 100-21-10) for 24 h, and then, activated HSCs were cocultured with SD-BMSCs by using 6-well Transwell inserts (0.4 µm, Corning, NY, United States, CAT: 3412), which were permeable to the culture medium but not cells. All coculture experiments were performed with HSCs seeded in the bottom chambers and BMSCs seeded in the insert chambers. After coculture for 12 h, the cells were washed twice with PBS, randomly divided into 5 groups and incubated in fresh DMEM. One group with normal medium was set as the model group. The group with medium containing 6 µg/ml colchicine was used as a positive control. Another three groups with medium with three doses of FA (1 mg/ml, 0.5 mg/ml, 0.25 mg/ml) were set as the FA groups. In addition, inactivated HSCs cocultured with BMSCs were used as the normal group. After coculture for 24 h, we collected HSCs and medium for subsequent experiments.

### Cell Migration Experiments

The cocultured cells were divided into 6 groups (NC, model, positive, FA-1 mg, FA-0.5 mg, and FA-0.25 mg). The specific cell coculture conditions were the same as those in the above experiments. When activated HSCs were treated with FA (1 mg/ml, 0.5 mg/ml, 0.25 mg/ml) and colchicine (6 μg/ml) for 24 h, we washed the cells with PBS twice, collected HSCs and seeded equal numbers of cells into 24-well Transwell inserts (8 μm, Corning, CAT: 3422), which allowed cell migration. After culture for 12 h, the cells were fixed with 4% paraformaldehyde for 20 min and washed twice with PBS, and the residual HSCs on the surface membranes were removed. Then, the cells were stained with crystal violet (Solarbio, CAT: C8470-25g) for 10 min and washed twice with PBS again. We removed the membranes from the inserts and counted the cells on the membrane by microscopy.

### Cytoskeletal Inhibitor and Agonist Experiments

HSCs were activated with TGF-β1 (10 ng/ml) for 24 h, and then, activated HSCs and BMSCs were cocultured with 0.4 µm Transwell inserts for 12 h. When cells adhered to wells, the coculture system was divided into three groups, and we next added fresh medium. One group with 1 µM cytochalasin D (cytoskeleton inhibitor, purchased from Sigma, Germany, CAT: C2618) was set as the cytoskeletal inhibitor group, one with 10 µM angiotensin II (cytoskeleton agonist, purchased from Sigma, CAT: A9525) was set as the cytoskeletal agonist group, and the normal and model groups had blank medium. After 24 h of culture, we collected cells and medium for subsequent experiments.

### Transfection of miR-19b-3p Mimics and a miR-19b-3p Negative Fragment Into Hepatic Stellate Cells

To detect the effect of miR-19b-3p on HSC activity, we designed miR-19b-3p mimics (5′-UGU GCA AAU CCA UGC AAA ACU GA-3′; KeyGEN, CAT:KGmiRNA) on the miR-19b-3p sequence and negative controls (NC-d). HSCs were activated in 10 ng/ml TGF-β1 medium without FBS for 24 h, digested by trypsin, centrifuged and counted. The cells were randomly divided into a model group, mimic group and negative control group, and inactivated HSC-T6 cells were set as the normal group. Therefore, all cells were divided into 4 groups: normal, model, HSC + mimics (mimic), and HSC + mimics NC (NC-d). Transient transfection was performed at a concentration of 10 μg/ml, and the cells were mixed evenly with miRNAs. The mixtures were added to a 4 mm shock tube, and the volume was not more than 900 μL. Shock tubes were placed into the BTX tester, the preset conditions were selected, and the detected instrument had no short circuit. After transfection, the cell mixture was transferred to a 6-well plate and cultured for 24 h. Cells were observed and captured with inverted fluorescence microscope (Axio observer A1, Zeiss, Germany). We collected HSCs and medium for subsequent experiments. All mimic and inhibitor were tagged with Cy3 fluorescence markers based.

### Transfection of miR-19b-3p Mimics, Inhibitors, and Negative Controls Into Bone Marrow Mesenchymal Stem Cells

To detect the effect of miR-19b-3p on SD-BMSCs, when BMSCs were digested, centrifuged and counted, we selected SD-BMSCs in good condition and randomly divided them into the model group, mimic group, inhibitor group, FA combined mimic group (FA + mimic group), FA combined inhibitor group (FA + inhibitor group), and negative control group (NC-d and NC-s). A total of 10 μg/ml miRNAs (miR-19b-3p mimic 5′-UGU GCA AAU CCA UGC AAA ACU GA-3′, KeyGEN; miR-19b-3p inhibitor 5′-UCA GUU UUG CAU GGA UUU GCA CA-3′, KeyGEN; miR-mimic NC-d, KeyGEN; miR-inhibitor NC-s KeyGEN) was used for transient transfection. The miRNAs all carried Cy3 markers. The cells and miRNAs were evenly mixed and placed into a 4 mm shock tube with a volume less than 900 μL. The shock tube was placed in the machine. After transfection, the cell mixture was transferred to inserts of 0.4 µm Transwell 6-well culture plates and cocultured with activated HSC-T6 cells for 24 h. After 24 h, we added fresh medium to each group. The medium of the mimic group and inhibitor group was exchanged with fresh DMEM containing 10% FBS. The mimic + FA and inhibitor + FA groups had fresh medium containing 1 mg/ml FA and 10% FBS. We also used normal BMSCs cocultured with resting HSCs or activated HSCs as a normal control or model and added normal fresh medium. Cells were divided into 8 groups: normal, model, BMSC + mimics (mimic), BMSC + inhibitors (inhibitor), BMSC + mimics + FA (mimic + FA), BMSC + inhibitors + FA (inhibitor + FA), BMSC + mimics NC (NC-d), and BMSC + inhibitor NC (NC-s). The coculture lasted for another 24 h. After coculture, we collected HSCs and medium for subsequent experiments.

### Enzyme-Linked Immunosorbent Assay

We extracted the culture medium and centrifuged the medium (×10000g, 20 min) to remove the cell debris. Then, we detected the contents of COL1-A1 (Mlbio, Shanghai, China, CAT: ml058800) and α-SMA (Mlbio, CAT:ml038078) in the culture medium with enzyme-linked immunosorbent assay (ELISA) kits according to the manufacturer’s protocol. The 20× washing solution was diluted to 1×, and an appropriate volume was prepared. Standard wells, sample wells and blank wells were used, and each group was assayed 3 times. We added 50 μL of the standard substance at different concentrations to each standard well, 50 μL of culture supernatant to the sample wells and 50 μL of sample dilution to the blank well. Next, we added 100 μL of horseradish peroxidase (HRP)-labelled detection antibody to each well, sealed the reaction well with a plate membrane, incubated it at 37°C for 60 min, discarded the liquid, washed it with 350 μL of washing solution 5 times, added 50 μL of substrates A and B to each well, incubated it at 37°C for 15 min in the dark, and added 50 μL of stop solution to each well. After 15 min, the OD value of each well was measured at a wavelength of 450 nm, and the sample well concentration was calculated. All data are presented as the mean ± SEM of values from at least three repeated experiments.

### Cloning of Vector Constructs and Luciferase Reporter Assay

For cloning of vector constructs, Rat miR-19b-3p were predicted by database: http://mirwalk.umm.uni-heidelberg.de. The 3′ untranslated region (UTR) of rat TGF-βR2 from genomic DNA, containing binding sites for rat miR-19b-3p, was amplified by PCR. The primer sequences used for vector construction were as follows: forward, 5′-TTC​TAG​TTG​TTT​AAA​CGA​GCT​CGC​TAG​CCT​CGA​GCT​TTT​TCT​GGG​CAG​GCT​GGG​CCA​AGA​CTC​CG-3′, reverse, 5′-GCA​GCC​GGA​TCA​GCT​TGC​ATG​CCT​GCA​GGT​CGA​CAA​GTG​CTA​ACG​TTA​TGC​CGA​GCC​CCT​TCC​GC-3'. The PCR product was purified using an AccuPrep PCR purification kit (Tiangen, CAT: DP204), cut by the restriction enzymes XhoI and SalI, and then cloned into the pmirGLO vector (Promega, WI, United States). The vector constructs with the 3′UTR of TGF-βR2 were transformed into *Escherichia coli*, and then, plasmid DNA was extracted from transformed, ampicillin-resistant *E. coli* using an AccuPrep Plasmid Mini Extraction Kit (Tiangen, Beijing, China, CAT: DP103). The sequences of the miR-19b-3p-binding sites of the 3′UTR of TGF-βR2 were confirmed by sequencing analysis (KeyGEN). Mutant vectors lacking the miR-19b-3p-binding site were manufactured by KeyGEN (KeyGEN, CAT: KGplasmid). For the luciferase reporter assay, HSC-T6 cells were seeded in 24-well culture plates in culture medium without P/S 1 day before transfection. The HSCs were divided into 3 groups: pmirGLO vector group, TGF-βR2-WT group, and TGF-βR2-MUT group. Either 10 µg miR-19b-3p mimic (KeyGEN) or scrambled miRNA (NC-d; KeyGEN) was added to each group. The cell mixture was placed into a 4 mm shock tube with a volume less than 900 μL. The shock tube was placed in the machine. After transfection, the cell mixture was cultured in 6-well plates for 48 h. After transfection, the cells were collected and tested with the Dual-Luciferase reporter assay system (Beyotime, Shanghai, China, CAT:RG028). Then, 100 μL of lysate was added to each well. After the cells were fully lysed, centrifugation was performed at ×10000 g–×15000 g for 5 min, and the supernatant was taken for analysis. Firefly luciferase detection reagent and sea kidney luciferase detection buffer were dissolved, an appropriate amount of sea kidney luciferase detection buffer was taken according to the amount of 100 μL for each sample, and sea kidney luciferase detection substrate (100×) at 1:100 was added to prepare sea kidney luciferase detection working solution. According to the operating instructions of the instrument, we used a multifunctional microplate detector with a chemiluminescence detection function and set the determination interval to 2 s and the detection time to 10 s. For each sample, 50 μL was taken, 100 μL of firefly luciferase detection reagent was added, and the RLU was determined after blowing and mixing with a gun. The reporter lysate was used as a blank control. After completing the above steps for the determination of firefly luciferase activity, 100 μL of luciferase detection solution was added, the sample was blown and mixed, and the RLU was determined. With sea cucumber luciferase as an internal reference, the RLU value for firefly luciferase was divided by the RLU value for sea cucumber luciferase, and the activation degree of the target reporter gene in each sample was compared according to the obtained ratio. All firefly luciferase activity data were normalized to Renilla luciferase activity and are presented as the mean ± SEM of values from at least three repeated experiments.

### Quantitative Real-Time PCR Analysis

We took 20 mg frozen animal tissues/cell samples, added 400 μL of TRIzol, homogenized them twice for 5 s each time, fully ground them, and homogenized them at room temperature for 5–10 min. Next, we added 80 μL of chloroform, mixed the sample for 15 s, incubated the sample at room temperature for 10–15 min, and then centrifuged it at 4°C and ×12000 g for 15 min at high speed. After centrifugation, the solution was divided into 3 layers, and RNA was in the supernatant. The supernatant was carefully transferred to a new 1.5-ml centrifuge tube without RNA enzyme, isopropyl alcohol was added to an equal volume and mixed evenly, and the sample was incubated at room temperature for 10 min. The mixture was centrifuged again at ×25000 g and 4°C for 15 min. The supernatant was carefully removed, precipitated with 1.5 ml of 75% ethanol to remove the organic solvent, and incubated until the precipitate floated. The samples were centrifuged at ×25000 g at 4°C for 10 min. Ethanol was discarded, and the precipitate was dried at room temperature for 3 min. The precipitate was dissolved in 20 μL of RNA-free water. After mixing, the RNA concentration and quality were detected, and reverse transcription was conducted. After the isolated RNA was reverse transcribed into cDNA by using a reverse transcription kit (Tiangen Biotech, Beijing, China, CAT: KR201), we used quantitative PCR (qRT-PCR) with a SYBR Green Taq kit (KAPA Biotechnology, CAT: KK4601) on a 7500HT Fast real-time PCR system to assay target gene expression. The primers were designed using Primer 5 (Table 1). The PCR conditions were as follows: 1) 95°C for 30 s and 2) 40 cycles of 95°C for 20 s, 60°C for 20 s, and 72°C for 20 s. The experiment was repeated three times.

**TABLE 1:** Primer sequences.

Primer name	Sequences 5′-3′
GAPDH-F	CTG​GAG​AAA​CCT​GCC​AAG​TAT​G
GAPDH-R	GGT​GGA​AGA​ATG​GGA​GTT​GCT
ROCK1-F	CAA​AAA​TGA​TCA​GTG​GGC​TTG​G
ROCK1-R	CCT​ACA​AAA​GGT​AGC​TGA​TTG​CC
LIMK1-F	CAC​AGT​CAC​CCT​CGT​GTC​TAT​CC
LIMK1-R	TCT​CGT​CCA​GCG​GCA​CAT​T
RhoA-F	GAT​GGA​GCT​TGT​GGT​AAG​ACA​TGC
RhoA-R	GGC​TGT​CGA​TGG​AAA​AAC​ACA​TC
SRF-F	GCG​GCG​TTA​CAC​GAC​CTT​C
SRF-R	TCT​GAA​TCA​GCG​CCT​TGC​C
COL1-A1-F	CAC​CTA​CAG​CAC​GCT​TGT​GG
COL1-A1-R	GAT​TGG​GAT​GGA​GGG​AGT​TTA​C
α-SMA-F	ACC​CAG​GCA​TTG​CTG​ACA​G
α-SMA-R	AGA​AGC​ATT​TGC​GGT​GGA​C
U6-F	CTCGCTTCGGCAGCACA
U6-R	AAC​GCT​TCA​CGA​ATT​TGC​GT
miR-19b-F	CTC​AAC​TGG​TGT​CGT​GGA​GTC​GGC​AAT​TCA​GTT​GAG TCAGTTTT
miR-19b-R	ACA​CTC​CAG​CTG​GGT​GTG​CAA​ATC​CAT​GCA​A
Universal primer -A	TGGTGTCGTGGAGTCG
miR-29b-3p-F	CTC​AAC​TGG​TGT​CGT​GGA​GTC​GGC​AAT​TCA​GTT​GAG ACTGATTT
miR-29b-3p-R	ACA​CTC​CAG​CTG​GGT​AGC​ACC​ATT​TGA​A
miR-183-5p-F	CTC​AAC​TGG​TGT​CGT​GGA​GTC​GGC​AAT​TCA​GTT​GAG​CAG​TGA​AT
miR-183-5p-R	ACA​CTC​CAG​CTG​GGT​ATG​GCA​CTG​GTA​GAA​T

### Cytoskeleton Staining

HSCs were treated with TGFβ1 and then cocultured with BMSCs. After the coculture period, the cells were washed twice with PBS, fixed with 4% paraformaldehyde for 20 min, and permeabilized with 0.1% Triton X-100 in PBS for 2–3 min at room temperature. After the cells were washed with PBS twice, they were stained with phalloidin (1:200) (Keygen, CAT: KGMP001) for 30 min. After phalloidin staining, the cell nuclei were stained with DAPI (1:100) (Solarbio, CAT: C0060) for 10 min. The cells were observed and captured using a confocal laser scanning microscope (Zeiss SP8, Germany). We observed the cytoskeleton in the FITC channel (wavelength = 498 nm) and miR-19b-3p markers in the Cy3 channel (wavelength = 550 nm).

### Western Blot Analysis

Cells and liver tissues were lysed in RIPA buffer (Lablead, Beijing, China, CAT: P0013B) and fully ground for 20 min, and total protein was extracted. The protein concentration was measured using a BCA protein assay kit (Beyotime, Nanjing, China, CAT: P0012). Protein detection by Western blot analysis was performed routinely. Equal amounts of total protein were separated by 8% or 10% SDS-PAGE and transferred onto polyvinylidene fluoride (PVDF) membranes (0.45/0.22 µm, Milipore, United States, CAT: IPVH00010). The membranes were incubated with primary antibodies against α-SMA (1:1000; Sigma, CAT: A2547), Col1α1 (1:1000; Abcam, United States, CAT: ab260043), GAPDH (1:1000; Cell Signaling Technology, Boston, United States), ROCK (1:1000; CST, CAT: 4035S), RhoA (1:1000; CST, CAT: 2117T), LIMK1 (1:1000, CST, CAT: 3842S), SRF (1:1000, CST, CAT: 5147S), and TGF-βR2 (1:1000, CST, CAT: 79424T) overnight at 4°C. Then, an HRP-conjugated goat anti-rabbit IgG (1:10000; Jackson ImmunoResearch, United States, CAT: 312-005-003) secondary antibody was incubated at 37°C for 1 h. The immunobands were visualized using a chemiluminescent HRP substrate (NCM, Beijing, China, CAT: P10200). Finally, the immunoreactive protein bands were detected and imaged by the Image Lab system (Bio-Rad ChemiDoc XRS, United States; Fusion FX, Vilber, France). Protein expression was standardized to that of GAPDH and quantified using Gel-Pro Analyzer software (Media Cybernetics, Rockville, MD, United States).

### Statistical Analysis

The results are expressed as the mean ± SEM. Statistical differences were determined by one-way ANOVA and two-way ANOVA (SPSS 20.0, Chicago, IL, United States). *p* values < 0.05 were considered statistically significant.

## Results

### Ferulic Acid Promotes the Ability of Bone Marrow Mesenchymal Stem Cells to Decrease Serum Aspartate Aminotransferase and Alanine Aminotransferase Levels in Liver Fibrosis Models

**FIGURE 1:**
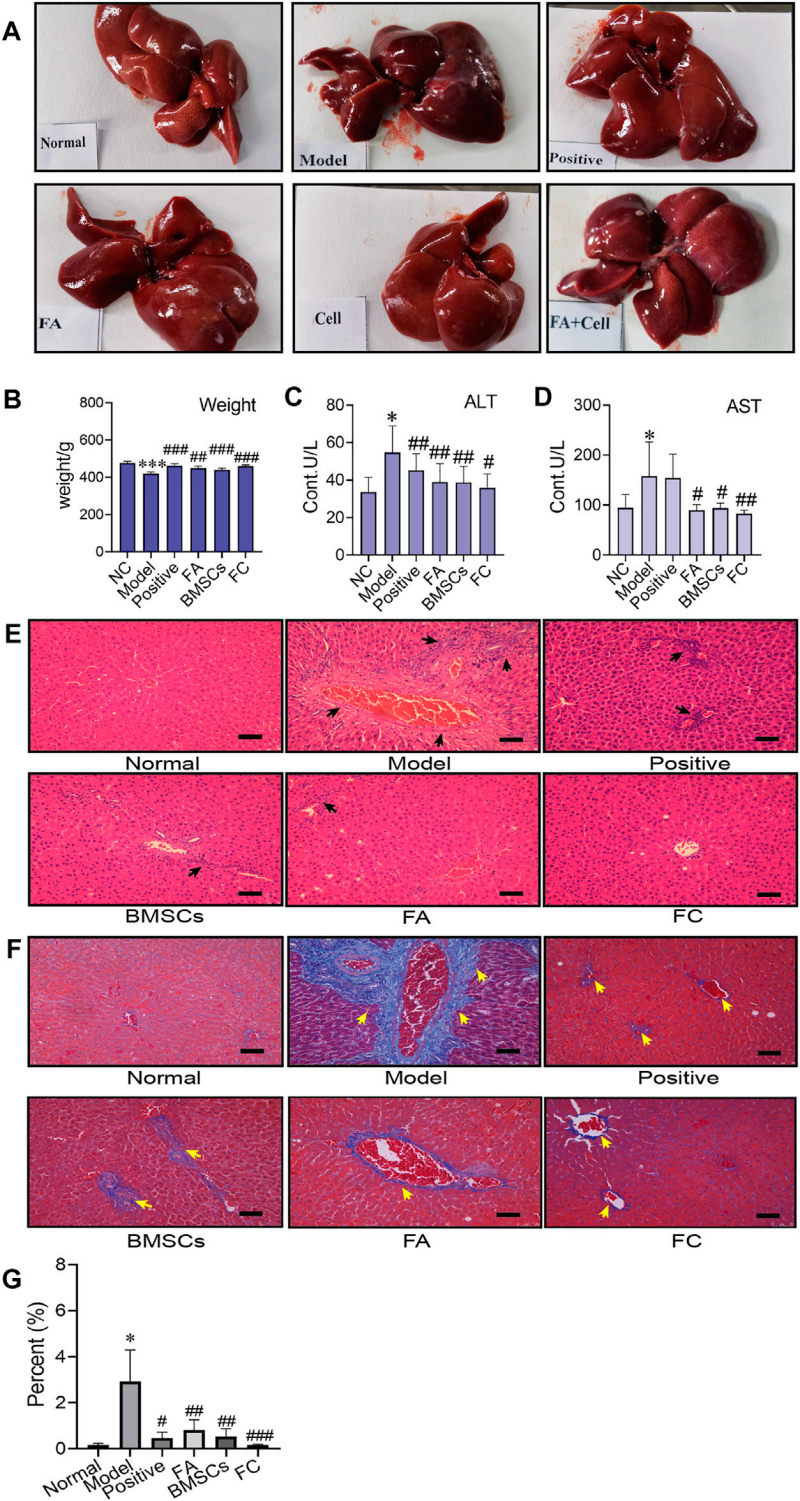
Administration of BMSCs combined with FA ameliorates CCl_4_-induced liver injury in rats. **(A)** The liver appearance of each group (normal, model, positive, BMSC, FA, and FC groups). **(B)** Comparison of body weight among the groups (Two-way ANOVA; *p < 0.05, **p < 0.01, ***p < 0.001 versus the normal group; #*p* < 0.05, ##*p* < 0.01, ###*p* < 0.001 versus the model group). **(C**,**D)** Serum levels of AST and ALT in each group (*n* = 6). Data are presented as the mean ± SEM (Two-way ANOVA; *p < 0.05 versus the normal group; #*p* < 0.05, ##*p* < 0.01 versus the model group). **(E)** Images of HE-stained liver sections from representative animals in the normal, model, positive, BMSC, FA, and FC groups (original magnification, ×10; scale bars, 100 µm, *n* = 6). **(F)** Masson stained liver sections from representative animals in the normal, model, positive, BMSC, FA, and BMSC&FA groups (original magnification, ×10; scale bars, 100 μm). The yellow squares indicate collagen. **(G)** The percentage of collagen area of Masson staining in each group (*n* = 6). Data are presented as the mean ± SEM (Two-way ANOVA; *p < 0.05 versus the normal group; #*p* < 0.05, ##*p* < 0.01, ###*p* < 0.001 versus the model group).

As shown in Figure 1A, the liver was dark red, the liver surface was rough, and adhesions appeared between the liver lobes in the CCl_4_-treated group compared to the normal group. In contrast, following BMSC and FA treatment, the liver changed substantially. The colour of the liver returned to light red, the liver surface was smooth, and adhesions were reduced in the BMSC + FA group compared to the model group. Rat weight also showed differences among the groups. Compared to that of the normal control rats, the weight of the model rats was significantly decreased (*p* < 0.001). The weight was significantly increased with FA and BMSC treatment (*p* < 0.001, vs. the model group), and there were no significant differences among the three treatment groups (Figure 1B). The serum ALT and AST levels in the model rats were significantly increased (*p* < 0.05, vs. control group), indicating successful establishment of the rat liver fibrosis model. FA, BMSC or FA&BMSC administration significantly decreased the serum AST and ALT levels (*p* < 0.05, vs. the model group) and there were no significant differences among the three treatment groups (Figures 1C,D).

### Histopathological Assessment of Liver Damage

To further confirm the antifibrotic effects of BMSCs, FA and their combination in an *in vivo* rat liver fibrosis model, we performed HE and Masson staining. As shown in Figures 1E,F, inflammatory cell infiltration, fragmented hepatic nuclei, and collagenous fibre formation were observed in the model group. Damage caused by CCl_4_ was significantly attenuated by BMSCs, FA, and the combination. In addition, the combination treatment achieved better reductions in collagenous fibres than the FA and BMSC single treatments.

### Ferulic Acid Promotes the Ability of Bone Marrow Mesenchymal Stem Cells to Improve Liver Fibrosis *via* the RhoA/ROCK Pathway

**FIGURE 2:**
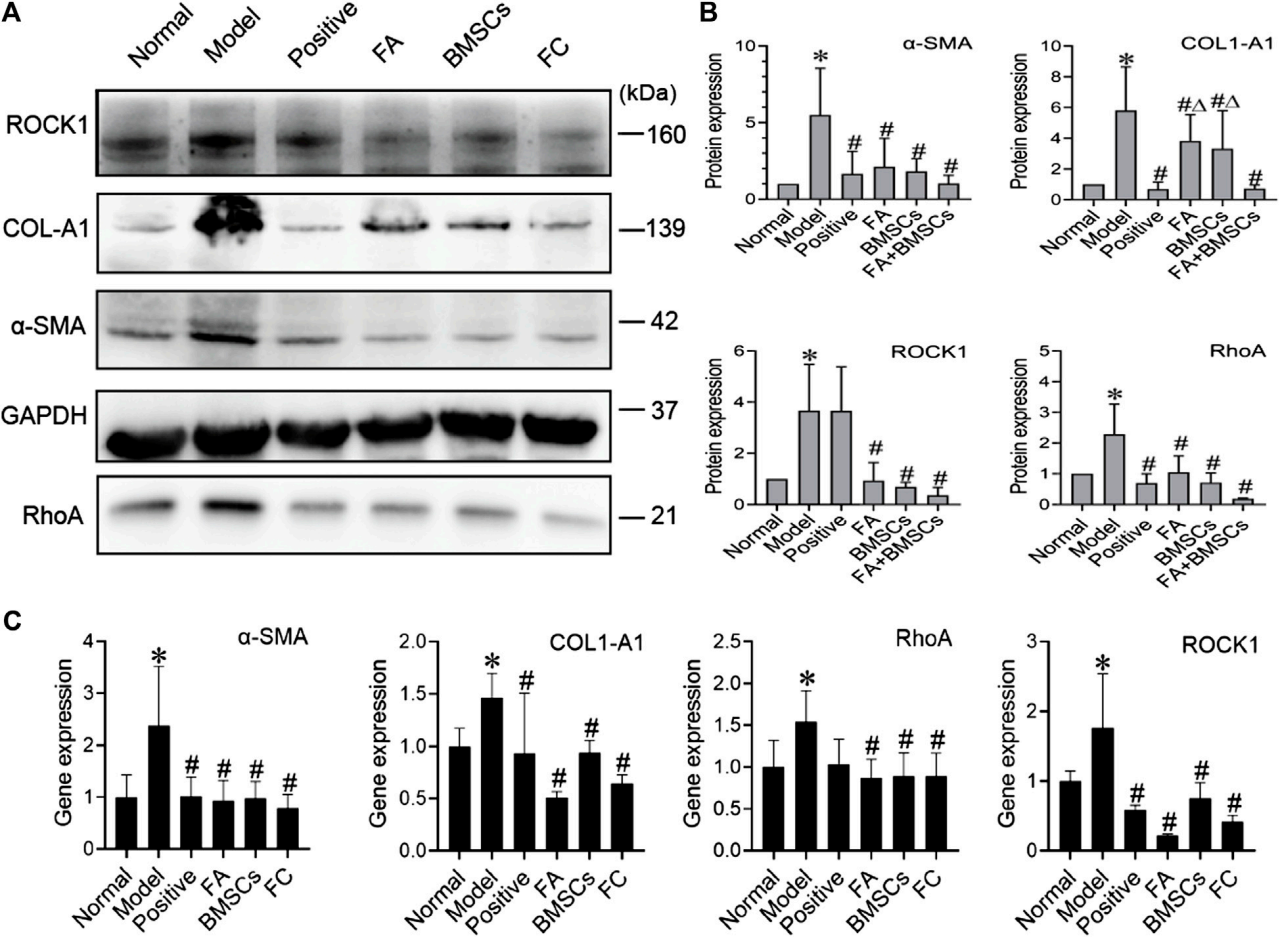
**(A**,**B)** Western blot and cumulative densitometric analyses of ROCK1 (160 kDa), COL1-A1 (139 kDa), α-SMA (42 kDa), GAPDH (37 kDa), and RhoA (21 kDa) in livers from each group (normal, model, positive, FA, BMSCs, FC). Data are presented as the mean ± SEM (*n* = 3 independent experiments, Two-way ANOVA; *p < 0.05 versus the normal group; #*p* < 0.05, versus the model group; Δp<0.05, versus the FC group); **(C)** qRT-PCR analysis for α-SMA, COL1-A1, ROCK1, and RhoA in rat livers. Data are presented as the mean ± SEM (*n* = 3 independent experiments, Two-way ANOVA; *p < 0.05 versus the normal group; #*p* < 0.05 versus the model group).

We found that FA could promote the ability of BMSCs to reduce liver fibrosis via the RhoA/ROCK pathway. Compared with that of the normal group, the protein expression of the model group showed significant increases in the profibrotic markers α-SMA and COL1-A1, while in the FA and BMSC-treated groups, their expression levels were decreased. In addition, FA combined with BMSCs showed better effects than the other two treatments on reducing COL1-A1 expression (*p* < 0.05). The expression patterns of RhoA and ROCK showed the same trends (Figures 2A,B).

QRT-PCR assays confirmed the protein data, showing reductions in α-SMA, COL1-A1, RhoA and ROCK expression in the FA, BMSC and FA&BMSC (FC) groups. These results clearly indicated that BMSCs combined with FA could be a novel treatment for liver fibrosis (Figure 2C).

### Ferulic Acid Promotes the Ability of Bone Marrow Mesenchymal Stem Cells to Inhibit Cytoskeletal Rearrangement in Hepatic Stellate Cells

**FIGURE 3:**
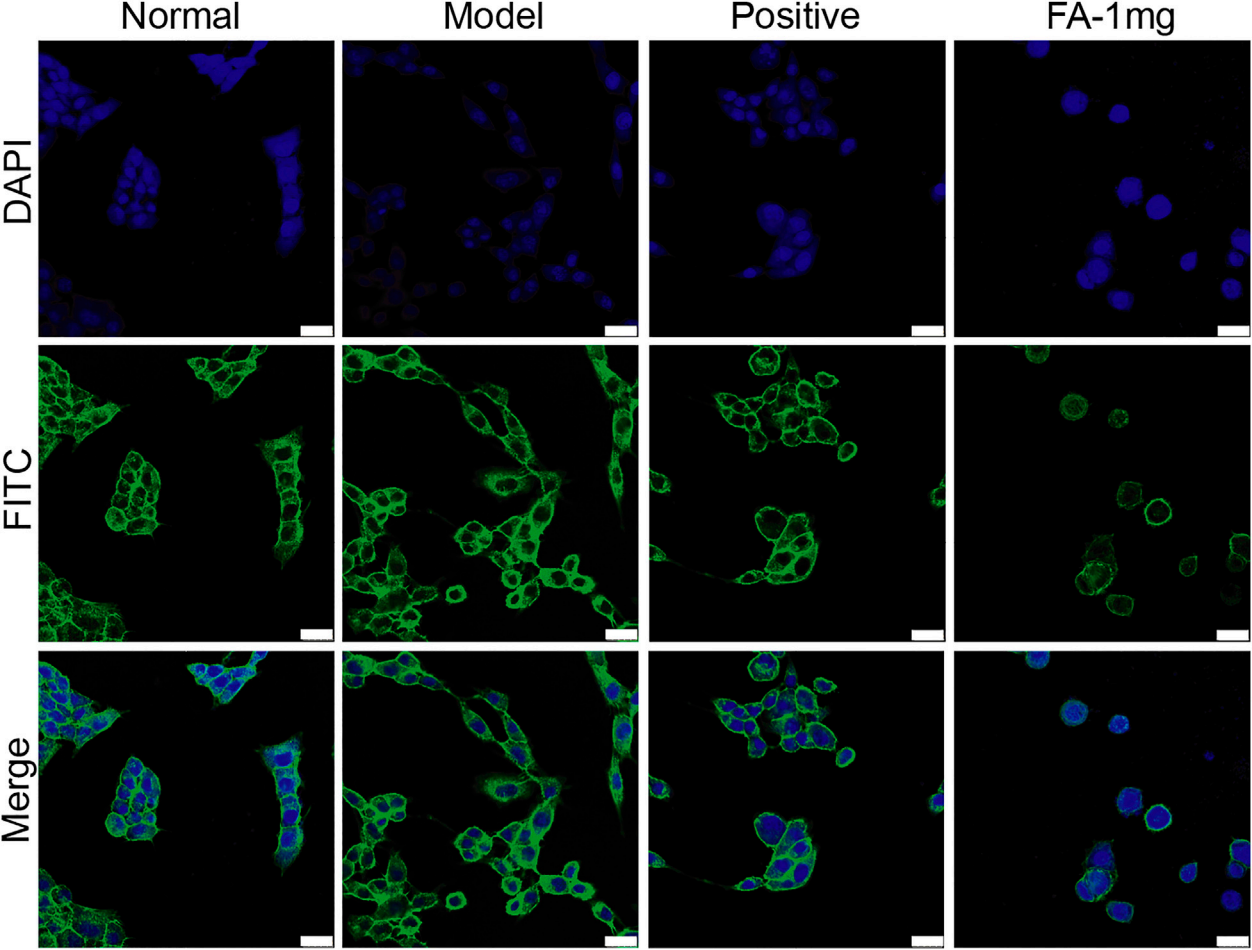
Representative images of the cytoskeleton (green) in the HSCs treated with BMSCs or BMSC&FA (1 mg/ml). DAPI was used to stain cell nuclei (original magnification, ×40; scale bars: 25 μm).

The activation of HSCs is an important factor in the formation of liver fibrosis. When stellate cells are activated, the cytoskeleton rearranges and promotes the migration of stellate cells. The RhoA/ROCK pathway is not only associated with the formation of fibrosis but also closely associated with cytoskeletal rearrangement. Therefore, we focused on whether FA promotes BMSC-mediated inhibition of cytoskeletal rearrangement in HSCs. Cocultured HSCs were evaluated by fluorescence and confocal microscopy and showed successful uptake. Compared to those in the normal group, activated HSCs exhibited F-actin remodelling to form a large number of thick stress fibres across the whole cell. In the FA-treated group, F-actin was observed in lamellipodia or filopodia in each cell, and a few fine stress fibres were found in these cells (Figure 3).

### Ferulic Acid Promotes Bone Marrow Mesenchymal Stem Cell-Mediated Inhibition of the Activation of Hepatic Stellate Cells *via* the RhoA/ROCK/SRF and RhoA/ROCK/LIMK1 Pathways

**FIGURE 4:**
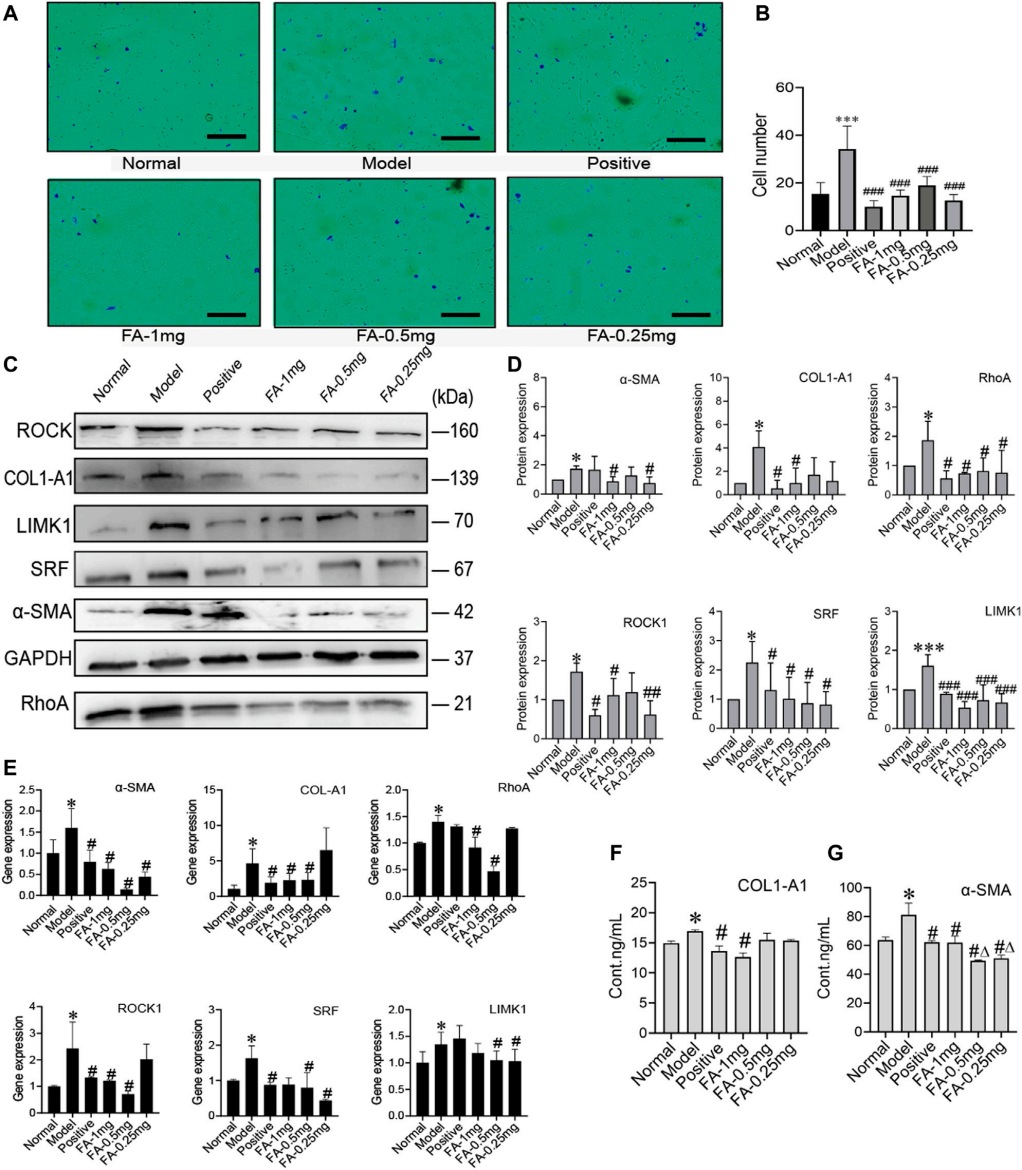
FA promotes BMSC-mediated inhibition of the activation of HSCs. **(A)** Migration of HSCs (original magnification, ×10; scale bars, 100 μm). **(B)** The number of migrating HSCs. (*n* = 3 independent experiments, Two-way ANOVA; ***p < 0.001 versus the normal group; ###*p* < 0.001, versus the model group); **(C**,**D)** Western blot bands and cumulative densitometric analyses of each group (normal, model, positive, FA-1 mg, FA-0.5 mg, FA-0.25 mg). (*n* = 3 independent experiments, Two-way ANOVA; *p < 0.05, ***p < 0.001, versus the normal group; #*p* < 0.05, ###*p* < 0.001, versus the model group); **(E)** qRT-PCR analysis for α-SMA, COL1-A1, ROCK1, RhoA, SRF, and LIMK1 in HSCs. (*n* = 3 independent experiments, Two-way ANOVA; *p < 0.05 versus the normal group; #*p* < 0.05 versus the model group); **(F**,**G)** The protein content of α-SMA and COL1-A1 in extracellular medium. *n* = 3 independent experiments, Two-way ANOVA; *p < 0.05 versus the normal group; #*p* < 0.05 versus the model group; Δp<0.05 versus the 1 mg FA group.

To investigate the promotive effect of FA on BMSC-mediated inhibition of HSC activation, we cultured activated HSC-T6 cells with an equal proportion of BMSCs as the model group, and activated HSC-T6 cells cocultured with BMSCs and treated with different doses of FA (1 mg/ml, 0.5 mg/ml, or 0.25 mg/ml FA) were used as the treatment groups. Inactivated HSCs cocultured with BMSCs in normal medium were used for the normal control group (NC group). We first detected the migration of HSCs. The activated HSC has great migration ability, the more active cells are, the more cells migrate in same period of time. The number of migration cell respects migration ability. HSC migration was significantly increased in the model group (*p* < 0.05, vs. the control group), but after FA treatment, HSC migration was significantly decreased (*p* < 0.05, vs. the model group), and 1 mg/ml FA showed a strong inhibitory effect (Figures 4A,B).

Western blot analysis confirmed that FA could promote BMSC-mediated inhibition of HSC activation, as indicated by assessment of HSC activation markers, including α-SMA and collagen type 1 a 1 chain (COL1-A1). The protein levels of α-SMA and COL1-A1 in the model group were significantly increased (*p* < 0.05, vs. the NC group). However, the expression of α-SMA and COL1-A1 was significantly decreased after FA treatment, and 1 mg/ml FA showed better and stable effects (*p* < 0.05, vs. the model group). We also detected α-SMA and COL1-A1 in extracellular medium by ELISA kits and found that their levels were decreased (Figures 4C,D). After confirming the effect of FA on HSC inactivation, we further assessed the direct pathway between BMSCs and HSCs. The cytoskeleton changed when HSCs were activated. We investigated the RhoA/ROCK/SRF and RhoA/ROCK/LIMK1 pathways. The protein levels of RhoA, ROCK, SRF, and LIMK1 were significantly increased in the model group compared to the NC group (*p* < 0.05), and after 1 mg/ml FA treatment, the expression of RhoA, ROCK, SRF, and LIMK1 was significantly decreased. Three dose groups showed no differences among them. The content of COL1-A1 and α-SMA in the ECM also decreased with 1 mg/ml FA treatment (Figures 4F,G). The mRNA levels showed the same trends (Figure 4E).

### Bone Marrow Mesenchymal Stem Cells Regulate the Activation of Hepatic Stellate Cells by Inhibiting Cytoskeletal Rearrangement

**FIGURE 5:**
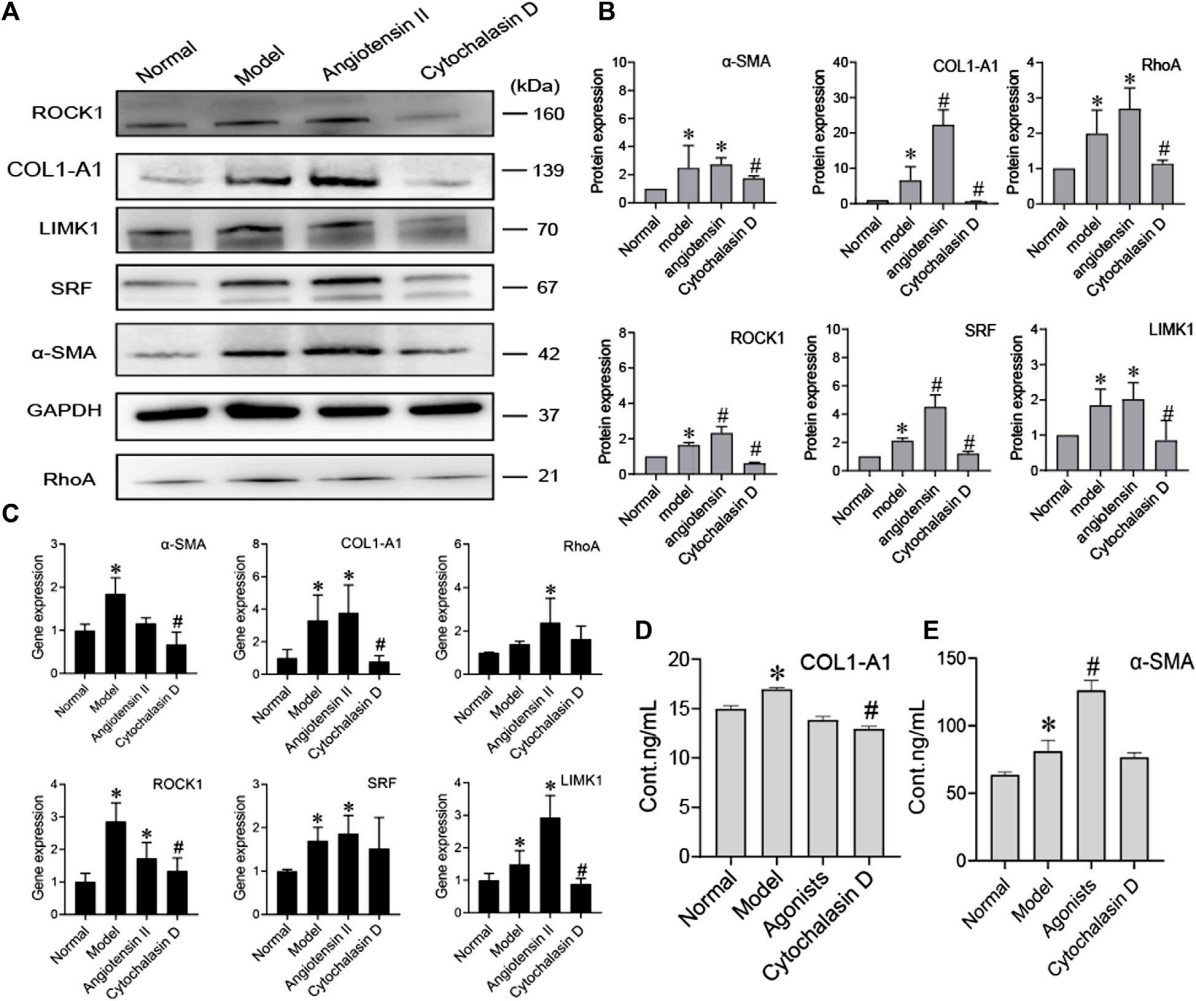
BMSCs regulate the activation of HSCs by inhibiting cytoskeletal rearrangement. **(A**,**B)** Western blot bands and cumulative densitometric analyses of each group (normal, model, angiotensin II, cytochalasin D). Data are presented as the mean ± SEM (*n* = 3 independent experiments, one-way ANOVA; *p < 0.05, versus the normal group; #*p* < 0.05, versus the model group); **(C)** qRT-PCR analysis for α-SMA, COL1-A1, ROCK1, RhoA, SRF, and LIMK1 in HSCs. (*n* = 3 independent experiments, one-way ANOVA; *p < 0.05 versus normal; #*p* < 0.05 versus model); **(D**,**E)** The protein content of α-SMA and COL1-A1 in extracellular medium. *n* = 3 independent experiments, one-way ANOVA; *p < 0.05 versus the normal group; #*p* < 0.05 versus the model group.

To further prove that the cytoskeleton pathway is directly involved in BMSC inhibition of HSC activation, we added a cytoskeleton inhibitor (cytochalasin D) or cytoskeleton agonist (angiotensin II) when BMSCs were cocultured with activated HSCs. Compared to the model group, the agonist group showed significantly increased protein expression of COL1-A1, α-SMA, RhoA, ROCK, SRF, and LIMK1 (*p* < 0.05), while the expression levels of these proteins were significantly decreased in the inhibitor group (*p* < 0.05) (Figures 5A,B). The content of COL1-A1 and α-SMA in the ECM also decreased with cytochalasin D treatment (Figures 5D,E). The RNA levels of COL1-A1, α-SMA, RhoA, ROCK, SRF, and LIMK1 were reduced in the cytochalasin D groups (Figure 5C). These results indicate that FA promotes the ability of BMSCs to influence HSC activation. The cytoskeleton-related RhoA/ROCK pathway directly interacts with BMSCs and HSCs.

### MiR-19b-3p Derived From Bone Marrow Mesenchymal Stem Cells Inactivates Hepatic Stellate Cells by Suppressing the Expression of its Target TGF-β Receptor II

**FIGURE 6:**
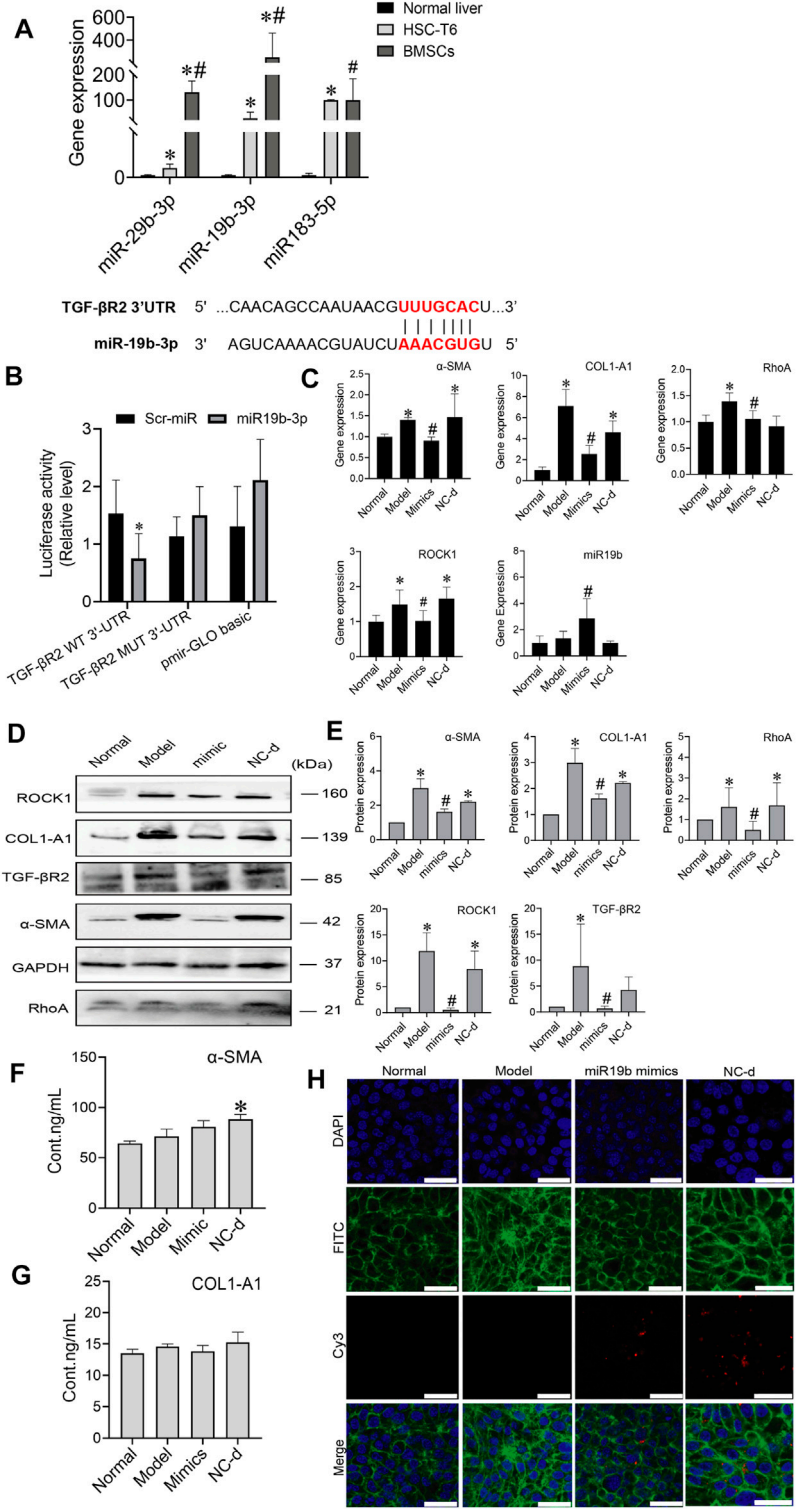
MiR-19b-3p derived from BMSCs inactivates HSCs by suppressing the expression of its target TGF-βR2. **(A)** The potential binding site (red fonts) of miR-19b-3p was predicted in the 3′UTR of TGF-βR2 mRNA in rats. The dashed line represents complementary base pairs between miR-19b-3p and TGF-βR2 mRNA. **(B)** A dual-luciferase assay was performed to verify the binding interaction between miR-19b-3p and TGF-βR2 mRNA. Cells cotransfected with pmirGLO basic vector containing either wild-type (WT) or mutant (mut) target sites plus either the miR-19b-3p mimic or scrambled (Scr-) miR (control); **(C)** qRT-PCR analysis for α-SMA, COL1-A1, ROCK1, and RhoA in HSCs. *n* = 3 independent experiments, one-way ANOVA; *p < 0.05 versus the normal group; #*p* < 0.05 versus the model group; **(D**,**E)** Western blot bands and cumulative densitometric analyses of each group (normal, model, mimic, NC-d). Data are presented as the mean ± SEM (*n* = 3 independent experiments, one-way ANOVA; *p < 0.05, versus the normal group; #*p* < 0.05, versus the model group). **(F**,**G)** The protein content of α-SMA and COL1-A1 in extracellular medium. *n* = 3 independent experiments, one-way ANOVA; *p < 0.05 versus the normal group; #*p* < 0.05 versus the model group. **(H)** Representative images of the cytoskeleton (green) in the HSCs treated with miR19b-3p mimics or NC-d. DAPI was used to stain cell nuclei (original magnification, ×40; scale bars: 25 μm).

MiRNAs are found in BMSCs, and miRNAs transferred into target cells can impact cell behaviours by regulating the expression of target genes. MiRNAs derived from BMSCs influenced HSC activation, we investigated which miRNAs contained in BMSCs regulated HSC activation. Three candidate miRNAs, miR-29b-3p, miR-19b-3p, and miR-183-5p, were selected because they were potentially linked to fibrosis in previous reports, including a microarray analysis (GEO: GSE168712) and small RNA sequencing analysis, as these three miRNAs are abundantly expressed in BMSCs. The levels of miR-29b-3p, miR-19b-3p, and miR-183-5p were higher in BMSCs than in HSCs or normal rat liver tissues, and among them, miR-19b-3p was significantly enriched in BMSCs, suggesting that miR-19b-3p might be involved in modulating HSC activation. Bioinformatic analysis using TargetScan and miRWalk predicted TGF-βR2, a receptor for TGF-β1, as a putative target of miR-19b-3p, and a luciferase reporter assay revealed that miR-19b-3p directly bound to the 3′UTR of the TGF-βR2 mRNA transcript (Figures 6A,B).

To determine whether miR-19b-3p affects HSC activation, we transfected HSCs with a miR-19b-3p mimic and a meaningless fragment (negative control). Additionally, we chose inactivated HSCs and activated HSCs as the normal and model controls, respectively. Although miR-19b-3p was rarely expressed in activated HSCs, its expression was strongly elevated in the miR-19b-3p-transfected HSCs (Figure 6C). In addition, the profibrotic markers α-SMA, COL1-A1, RhoA, and ROCK1 had downregulated expression in the HSCs transfected with the miR-19b-3p mimics compared with those in the model and negative control groups (*p* < 0.05) (Figures 6D,E). The content of α-SMA and COL1-A1 in the ECM did not show a decreasing trend in the mimic group (Figures 6F,G). Western blot assays confirmed the RNA data, showing reductions in TGF-βR2 and profibrotic marker expression in the HSCs transfected with miR-19b-3p. These results suggest that miR-19b-3p inhibits HSC activation by directly targeting TGF-βR2.

We also transfected the miR-19b-3p mimic, or negative control with the fluorescent Cy3 gene into activated HSCs. With confocal scanning microscopy, we observed red fluorescence in the miR-19b-3p mimic and negative control groups. Compared to the model group, the group transfected with the miR-19b-3p mimics showed F-actin mainly distributed in peripheral cells, and a few fine stress fibres were found in these cells. The cytoskeleton of cells in the negative control groups was similar to that of the cells in the model group (Figure 6H).

### Ferulic Acid Induces Bone Marrow Mesenchymal Stem Cells to Release More miR-19b-3p to Hepatic Stellate Cells and Inhibit the Activation of Hepatic Stellate Cells

**FIGURE 7:**
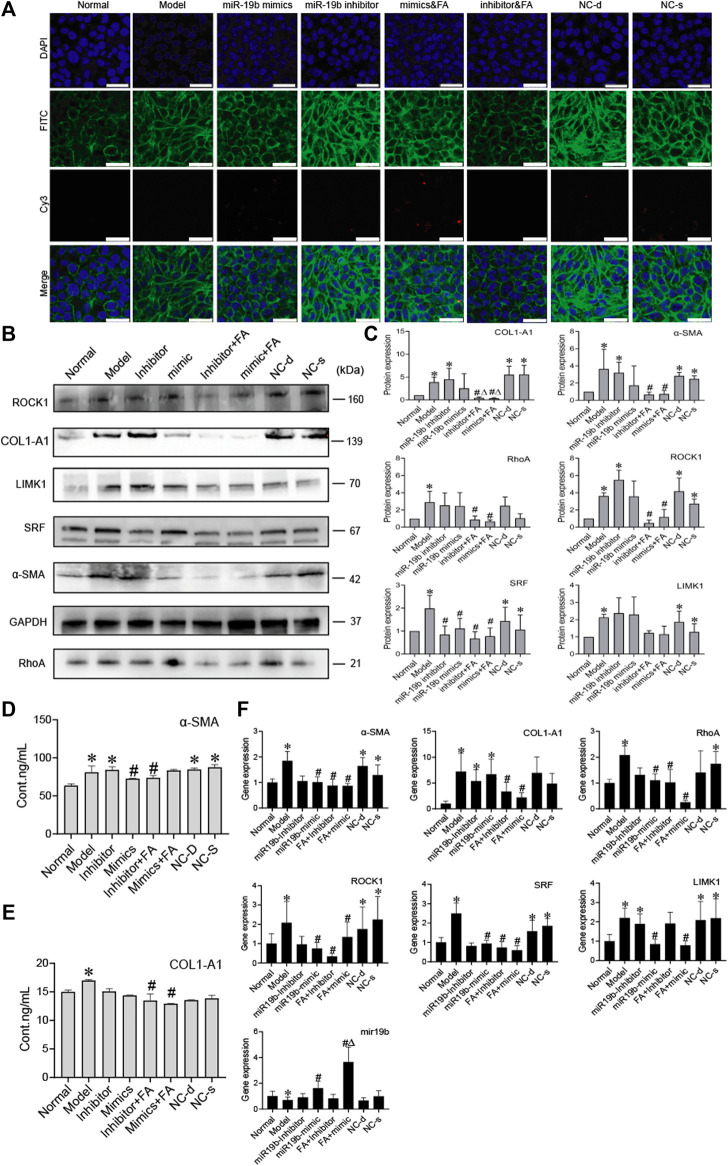
FA induces BMSCs to release more miR-19b-3p to HSCs and inhibit the activation of HSCs. **(A)** Representative images of the cytoskeleton (green) in the HSCs treated with miR19b-3p mimics, inhibitor, or NC-d and NC-s. DAPI was used to stain cell nuclei (original magnification, ×40; scale bars: 25 μm). **(B**,**C)** Western blot bands and cumulative densitometric analyses of each group (normal, model, inhibitor, mimic, inhibitor&FA, mimic&FA, NC-d, NC-s). Data are presented as the mean ± SEM (*n* = 3 independent experiments, Two-way ANOVA; *p < 0.05, versus the normal group; #*p* < 0.05, versus the model group; Δp < 0.05, versus the inhibitor/mimic group). **(D**,**E)** The protein content of α-SMA and COL1-A1 in extracellular medium. *n* = 3 independent experiments, Two-way ANOVA; *p < 0.05 versus the normal group; #*p* < 0.05 versus the model group; **(F)** qRT-PCR analysis for α-SMA, COL1-A1, ROCK1, RhoA, SRF, and LIMK1 in HSCs. *n* = 3 independent experiments, Two-way ANOVA; *p < 0.05 versus the normal group; #*p* < 0.05 versus the model group; Δp < 0.05 versus the miR19b mimic group.

To determine whether BMSCs transfer miR-19b-3p to HSCs, we transfected the miR-19b-3p mimic or miR-19b-3p inhibitor with the fluorescent Cy3 gene into BMSCs and cocultured the transfected cells with activated HSCs. With confocal scanning microscopy, we observed red fluorescence in the miR-19b-3p mimic group, and F-actin was mainly distributed in peripheral cells. A few fine stress fibres were found in these cells compared to those in the model group. In addition, in the miR-19b-3p inhibitor group, the cell shape was similar to that in the model group, and F-actin in activated HSCs was remodelled to form a large number of thick stress fibres across the whole cell. With FA treatment, we found more red fluorescence in the miR-19b-3p mimic groups (Figure 7A).

To assess protein levels, we divided the BMSC/HSC coculture system into 8 groups: the normal, model, mimic, inhibitor, mimic + FA, inhibitor + FA, and negative control groups. We detected α-SMA, COL1-A1, RhoA, ROCK1 SRF, and found that their expression was significantly downregulated in the mimic + FA, and inhibitor + FA groups compared to the model group, while mimic + FA, and inhibitor + FA groups also showed significance respectively compared to mimics/inhibitor group on COL1-A1 expression. The mimic group showed the decreased trends (Figures 7B,C). The content of α-SMA and COL1-A1 in the ECM also showed a decreasing trend in the mimic, inhibitor&FA, mimic&FA group (Figures 7D,E). The mRNA levels showed the same trends (Figure 7F).

These results suggest that miR-19b-3p plays a critical role in regulating the effect of BMSCs against liver fibrosis. In conclusion, these results suggest that FA combined with BMSC treatment can induce greater release of miR-19b-3p, which inhibits the activation of HSCs by suppressing the RhoA/ROCK1/SRF and RhoA/ROCK1/LIMK1 pathways, contributing to alleviation of liver fibrosis.

## Discussion

In this study, we showed that FA combined with BMSCs was more effective in treating liver fibrosis than single treatment. We used BMSCs, FA or combination therapy to treat rats with hepatic fibrosis. By evaluating the AST, ALT, HE, and Masson staining results, we observed that liver fibrosis was decreased to different degrees and that the combination therapy showed better effects than FA or BMSC monotherapy. In addition, we detected the protein expression of COL1-A1, α-SMA, ROCK1 and RhoA, and the results for the combination therapy showed better effects. Therefore, these results suggest that FA can promote the ability of BMSCs to improve liver fibrosis by targeting the RhoA/ROCK pathway.

The RhoA/ROCK signalling pathway is related to intracellular cytosis, colonization, migration, apoptosis, gene expression and other behaviours (Xu et al., 2018; Yaqoob et al., 2020). The RhoA/ROCK pathway also affects the formation of fibrosis. This axis promotes collagen and ECM production in fibroblasts and increases the transformation of fibroblasts into myofibroblasts expressing α-SMA and other activated factors (Deng et al., 2019; Sloniecka and Danielson 2019). RhoA/ROCK is a classic fibrosis pathway, which has quantities of related studies have been carried out as a preliminary basis, and ROCK1 is also associated with cytoskeleton and cytodynamics. Therefore, we choose RhoA/ROCK pathway to study.

To verify the mechanism of BMSC and FA combination therapy, we established a BMSC/HSC-T6 coculture system. Activated HSCs were cocultured with an equal amount of BMSCs. We observed the cellular structure of HSC-T6 cells and found that with FA treatment, the deformed cell structure was restored, F-actin was mainly distributed in peripheral cells, and a few fine stress fibres were found in these cells. In addition to assessing cellular structure, we detected the migration of HSCs. The number of migrating cells was significantly decreased with FA and BMSC treatment. The protein expression of RhoA, ROCK, SRF, LIMK1, COL1-A1, and α-SMA was significantly decreased. These results all proved that FA promoted the ability of BMSCs to decrease HSC migration while inhibiting activation.

The RhoA/ROCK pathway is related to both development of fibrosis and cytoskeletal rearrangement. RhoA/ROCK/SRF directly influences collagen and α-SMA production, while RhoA/ROCK/LIMK1 influences cytoskeletal rearrangement, indirectly affecting HSC migration. Activated HSCs migrate to sites of damage and release collagen. Therefore, inhibiting cytoskeletal rearrangement is also an effective strategy for reducing fibrosis (Ni et al., 2013; Wu et al., 2019).

To verify the mechanism by which BMSCs inhibit HSC activation, we established a BMSC/HSC coculture system with angiotensin II and cytochalasin D used as agonists and inhibitors, respectively. The decreased protein expression of RhoA, ROCK, SRF, LIMK1, COL1-A1, and α-SMA also indicated that BMSCs could reduce HSC activation by inhibiting cytoskeletal rearrangement.

Many studies have proven that BMSCs can produce miRNAs (Cetin et al., 2021). Feng et al. demonstrated that MSC-derived miRNAs reduced infarct size and cardiac fibrosis by inhibiting apoptosis in mice with myocardial infarction. Exosomes derived from chorionic plate-derived MSCs (CP-MSCs) containing miR-125b could ameliorate hepatic fibrosis by regulating hedgehog (Hh) signalling, which is an essential regulator in liver fibrosis (Feng et al., 2014; Hyun et al., 2015). These findings suggest that miRNAs play important roles in MSC-mediated tissue repair and regeneration. In a recent study, BMSCs were found to release miRNAs, targeting specific proteins to reduce fibrosis (Chen et al., 2018; BI Hui-yang 2019). The results for the BMSC genomic sequence showed that BMSCs contain a variety of miRNAs (Lindoso et al., 2014; Zhao et al., 2017). Among them, miR-19b-3p was shown to be related to inhibition of fibrosis (Zou et al., 2016; Chen et al., 2019; Duan et al., 2019; Wang et al., 2021). We used the TargetScan website to predict the target genes of miR-19b-3p and found TGF-βR2 was a putative target. The luciferase reporter assay revealed that miR-19b-3p directly bound to TGF-βR2 in HSCs.

To determine whether miR-19b-3p can inhibit HSC activation, we transfected miR-19b-3p mimics or inhibitors with the Cy3 gene into HSCs and BMSCs. We observed the cytoskeleton of the HSCs transfected with miR-19b-3p mimics or inhibitors. With miR-19b-3p mimic transfection, the cytoskeleton of activated HSCs was not substantially changed, while with miR-19b-3p inhibitor transfection, the cytoskeleton of activated HSCs was still rearranged. The decreased expression of RhoA, ROCK, SRF, LIMK1, COL1-A1 and α-SMA showed that miR-19b-3p could affect HSC activation.

We also observed that the BMSCs transfected with the mimics released more miR-19b-3p to HSCs and that the cytoskeleton was similar to that of inactivated HSCs; the BMSCs transfected with the inhibitors released less miR-19b-3p to HSCs, and the cytoskeleton was similar to that of activated HSCs. The protein expression of RhoA, ROCK, SRF, LIMK1, COL1-A1, and α-SMA showed that BMSCs could release miR-19b-3p to inhibit HSC activation, and BMSCs combined with FA treatment resulted in better HSC inhibition. These results indicate that combining BMSCs with FA could be a promising therapeutic strategy for the treatment of liver disease.

Bone marrow is the most widely used source of BMSCs. However, due to the instability of BMSCs, the use of these cells in clinical applications is limited (Alsulami and Abdel-Gaber 2021; Muslimah Alsulami, 2021; Yang et al., 2021). FA is a derivative of cinnamic acid and has therapeutic activity against a variety of diseases. This molecule is also an effective ingredient in some TCMs, such as *Angelica sinensis*. In addition, FA may protect against fibrotic deterioration in rats with hepatic fibrosis (Mu et al., 2018). Therefore, we chose FA combined with BMSCs to treat hepatic fibrosis in rats and found that their combination showed better treatment effects.

## Conclusion

Collectively, we demonstrated that FA promoted the ability of BMSCs to suppress HSC activation and liver fibrosis. MiR-19b-3p derived from BMSCs inactivated HSCs by suppressing the RhoA/ROCK/SRF and RhoA/ROCK/LIMK1 signalling pathways. Therefore, our findings suggest that BMSCs combined with a TCM have potential as an effective antifibrotic drug for chronic liver diseases.

## Data Availability

The original contributions presented in the study are included in the article/Supplementary Material, further inquiries can be directed to the corresponding author.

## Abbreviations

SD rat, Sprague-Dawley rat; ANOVA, analysis of variance; DAPI, diamidinophenylindole; DMEM, Dulbecco’s modified Eagle’s medium; FA, ferulic acid; FBS, foetal bovine serum; PBS, phosphate-buffered saline; BMSCs, bone marrow mesenchymal stem cells; HE, haematoxylin and eosin; ROCK1, Rho-associated kinase 1; RhoA, Ras homologue family member A; SRF, serum response factor; LIMK1, LIM domain kinase 1; COL1-A1, collagen type I alpha 1 chain; α-SMA, alpha-smooth muscle actin; TGF-β1, transforming growth factor beta 1; TGF-βR2, transforming growth factor beta receptor 2; ECM, extracellular matrix; HSCs, hepatic stellate cells.

## References

[B1] Alfaifi M. Eom Y. W. Newsome P. N. Baik S. K. (2018). Mesenchymal Stromal Cell Therapy for Liver Diseases. J. Hepatol. 68 (6), 1272–1285. 10.1016/j.jhep.2018.01.030 29425678

[B2] Ali S. A. Saifi M. A. Pulivendala G. Godugu C. Talla V. (2021). Ferulic Acid Ameliorates the Progression of Pulmonary Fibrosis via Inhibition of TGF-Β/smad Signalling. Food Chem. Toxicol. 149, 111980. 10.1016/j.fct.2021.111980 33428986

[B3] Alsulami M. Abdel-Gaber R. (2021). Cell Therapy as a New Approach on Hepatic Fibrosis of Murine Model of Schistosoma Mansoni-Infection. Acta Parasitol. 66 (1), 136–145. 10.1007/s11686-020-00267-2 32816183

[B4] Alsulami M. Abdel-Gaber R. (2021). Cell Therapy as a New Approach on Hepatic Fibrosis of Murine Model of Schistosoma Mansoni-Infection. Acta Parasit. 66, 136–145. 10.1007/s11686-020-00267-2 32816183

[B5] Ankrum J. Karp J. M. (2010). Mesenchymal Stem Cell Therapy: Two Steps Forward, One Step Back. Trends Mol. Med. 16 (5), 203–209. 10.1016/j.molmed.2010.02.005 20335067 PMC2881950

[B6] Bataller R. Brenner D. A. (2005). Liver Fibrosis. J. Clin. Invest. 115 (2), 209–218. 10.1172/jci20052428210.1172/JCI24282 15690074 PMC546435

[B7] Bi Hui-yang G. J-l. (2019) Research Progress of miRNA Related to Bone Marrow Mesenchymal Stem Cells. World Latest Medicine Information (Electronic Version) 19 (52), 68–69. doi:10.19613/j.cnki.1671-3141.2019.52.035

[B8] Cetin Z. Saygili E. I. Görgisen G. Sokullu E. (2021). Preclinical Experimental Applications of miRNA Loaded BMSC Extracellular Vesicles. Stem Cel Rev Rep 17 (2), 471–501. 10.1007/s12015-020-10082-x 33398717

[B9] Chen L. Lu F. B. Chen D. Z. Wu J. L. Hu E. D. Xu L. M. (2018). BMSCs-Derived miR-223-Containing Exosomes Contribute to Liver protection in Experimental Autoimmune Hepatitis. Mol. Immunol. 93, 38–46. 10.1016/j.molimm.2017.11.008 29145157

[B10] Chen W. Zhou Z. Q. Ren Y. Q. Zhang L. Sun L. N. Man Y. L. (2019). Effects of Long Non-coding RNA LINC00667 on Renal Tubular Epithelial Cell Proliferation, Apoptosis and Renal Fibrosis via the miR-19b-3p/LINC00667/CTGF Signaling Pathway in Chronic Renal Failure. Cell Signal 54, 102–114. 10.1016/j.cellsig.2018.10.016 30555030

[B11] Deng Z. Jia Y. Liu H. He M. Yang Y. Li Y. (2019). RhoA/ROCK Pathway: Implication in Osteoarthritis and Therapeutic Targets. Am. J. Transl Res. 11 (9), 5324–5331. 31632513 PMC6789288

[B12] Duan L. Duan D. Wei W. Sun Z. Xu H. Guo L. (2019). MiR-19b-3p Attenuates IL-1β Induced Extracellular Matrix Degradation and Inflammatory Injury in Chondrocytes by Targeting GRK6. Mol. Cel Biochem 459 (1-2), 205–214. 10.1007/s11010-019-03563-2 31227976

[B13] Feng Y. Huang W. Wani M. Yu X. Ashraf M. (2014). Ischemic Preconditioning Potentiates the Protective Effect of Stem Cells through Secretion of Exosomes by Targeting Mecp2 via miR-22. PLoS One 9 (2), e88685–8. 10.1371/journal.pone.0088685 24558412 PMC3928277

[B14] Hyun J. Wang S. Kim J. Kim G. J. Jung Y. (2015). MicroRNA125b-mediated Hedgehog Signaling Influences Liver Regeneration by Chorionic Plate-Derived Mesenchymal Stem Cells. Sci. Rep. 5, 14135. 10.1038/srep14135 26370741 PMC4569897

[B15] Jayamani J. Naisini A. Madhan B. Shanmugam G. (2018). Ferulic Acid, a Natural Phenolic Compound, as a Potential Inhibitor for Collagen Fibril Formation and its Propagation. Int. J. Biol. Macromol 113, 277–284. 10.1016/j.ijbiomac.2018.01.225 29476853

[B16] Jingjing H. Hongna H. Wenfu Z. Jianlin L. Guochu H. Yuanjia L. (2021). Bie Jia Jian Pill Combined with Bone Mesenchymal Stem Cells Regulates microRNA-140 to Suppress Hepatocellular Carcinoma Stem Cells. Int. J. Stem Cell 14 (3), 275–285. 10.15283/ijsc20157 PMC8429943 33632990

[B17] Li D. Rui Y. X. Guo S. D. Luan F. Liu R. Zeng N. (2021). Ferulic Acid: A Review of its Pharmacology, Pharmacokinetics and Derivatives. Life Sci. 284, 119921. 10.1016/j.lfs.2021.119921 34481866

[B18] Li R. Luo X. Zhu Y. Zhao L. Li L. Peng Q. (2017). ATM Signals to AMPK to Promote Autophagy and Positively Regulate DNA Damage in Response to Cadmium-Induced ROS in Mouse Spermatocytes. Environ. Pollut. 231 (Pt 2), 1560–1568. 10.1016/j.envpol.2017.09.044 28964605

[B19] Liao L. Shi B. Chang H. Su X. Zhang L. Bi C. (2017). Heparin Improves BMSC Cell Therapy: Anticoagulant Treatment by Heparin Improves the Safety and Therapeutic Effect of Bone Marrow-Derived Mesenchymal Stem Cell Cytotherapy. Theranostics 7 (1), 106–116. 10.7150/thno.16911 28042320 PMC5196889

[B20] Lindoso R. S. Collino F. Bruno S. Araujo D. S. Sant'Anna J. F. Tetta C. (2014). Extracellular Vesicles Released from Mesenchymal Stromal Cells Modulate miRNA in Renal Tubular Cells and Inhibit ATP Depletion Injury. Stem Cell Dev 23 (15), 1809–1819. 10.1089/scd.2013.0618 PMC4103261 24669934

[B21] Mu M. Zuo S. Wu R. M. Deng K. S. Lu S. Zhu J. J. (2018). Ferulic Acid Attenuates Liver Fibrosis and Hepatic Stellate Cell Activation via Inhibition of TGF-β/Smad Signaling Pathway. Drug Des. Devel Ther. 12, 4107–4115. 10.2147/DDDT.S186726 PMC6284527 30584275

[B22] Ni J. Dong Z. Han W. Kondrikov D. Su Y. (2013). The Role of RhoA and Cytoskeleton in Myofibroblast Transformation in Hyperoxic Lung Fibrosis. Free Radic. Biol. Med. 61, 26–39. 10.1016/j.freeradbiomed.2013.03.012 23517783 PMC3849210

[B23] Pinzani M. Rombouts K. (2004). Liver Fibrosis: from the Bench to Clinical Targets. Dig. Liver Dis. 36, 231–242. 10.1016/j.dld.2004.06.00110.1016/j.dld.2004.01.003 15115333

[B24] Rong X. Liu J. Yao X. Jiang T. Wang Y. Xie F. (2019). Human Bone Marrow Mesenchymal Stem Cells-Derived Exosomes Alleviate Liver Fibrosis through the Wnt/β-Catenin Pathway. Stem Cel Res Ther 10 (1), 98. 10.1186/s13287-019-1204-2 PMC6421647 30885249

[B25] Rong X. Yang Y. Zhang G. Zhang H. Li C. Wang Y. (2020). Antler Stem Cells as a Novel Stem Cell Source for Reducing Liver Fibrosis. Cell Tissue Res 379 (1), 195–206. 10.1007/s00441-019-03081-z 31428875 PMC12660448

[B26] Słoniecka M. Danielson P. (2019). Substance P Induces Fibrotic Changes through Activation of the RhoA/ROCK Pathway in an *In Vitro* Human Corneal Fibrosis Model. J. Mol. Med. 97 (10), 1477–1489. 10.1007/s00109-019-01827-4 31399750 PMC6746877

[B27] Tian J. Z. H. W. M. S. L. X. J. Z. Y. T. L. T. H. L. P. (2016). Therapeutical Effect of Saponins fromRhizoma Panacis Majoris Combined with BMSCs on Hepatic Fibrosis Induced by CCl_4 in Rats. J. China Three gorges Univ. (Natural Science) 38 (3), 104–108. 10.13393/j.cnki.issn.1672-948X.2016.03.023

[B28] Virarkar M. Morani A. C. Taggart M. W. Bhosale P. (2021). Liver Fibrosis Assessment. Semin. Ultrasound CT MR 42 (4), 381–389. 10.1053/j.sult.2021.03.003 34130850

[B29] Wang Y. Wang L. Guo J. Zuo S. Wang Z. Hua S. (2021). MYPT1, Regulated by miR-19b-3p Inhibits the Progression of Non-small Cell Lung Cancer via Inhibiting the Activation of Wnt/β-Catenin Signaling. Life Sci. 278, 1–10. 10.1016/j.lfs.2021.119573 33964297

[B30] Wu Y. Li Z. Wang S. Xiu A. Zhang C. (2019). Carvedilol Inhibits Angiotensin II-Induced Proliferation and Contraction in Hepatic Stellate Cells through the RhoA/Rho-Kinase Pathway. Biomed. Res. Int. 2019, 1–15. 10.1155/2019/7932046 PMC6885148 31828132

[B31] Xu A. Li Y. Zhao W. Hou F. Li X. Sun L. (2018). PHP14 Regulates Hepatic Stellate Cells Migration in Liver Fibrosis via Mediating TGF-Β1 Signaling to PI3Kγ/AKT/Rac1 Pathway. J. Mol. Med. (Berl) 96 (2), 119–133. 10.1007/s00109-017-1605-6 29098317

[B32] Yang X. Chen Z. Chen C. Han C. Zhou Y. Li X. (2021). Bleomycin Induces Fibrotic Transformation of Bone Marrow Stromal Cells to Treat Height Loss of Intervertebral Disc through the TGFβR1/Smad2/3 Pathway. Stem Cel Res Ther 12 (1), 34. 10.1186/s13287-020-02093-9 PMC7791639 33413668

[B33] Yao X. Wang J. Zhu J. Rong X. (2020). The Anti-fibrotic Effect of Human Fetal Skin-Derived Stem Cell Secretome on the Liver Fibrosis. Stem Cel Res Ther 11 (1), 379. 10.21203/rs.3.rs-39850/v110.1186/s13287-020-01891-5 PMC7650526 32883340

[B34] Yaqoob U. Luo F. Greuter T. Jalan Sakrikar N. Sehrawat T. S. Lu J. (2020). GIPC-regulated IGFBP-3 Promotes HSC Migration In Vitro and Portal Hypertension In Vivo through a β1-Integrin Pathway. Cell Mol Gastroenterol Hepatol 10 (3), 545–559. 10.1016/j.jcmgh.2020.05.005 32447051 PMC7399184

[B35] Ye Chao W. Q. Chen J. Zheng Y. Qu Y. I. Wang J. Wang F. (2021). Repair Effect and Mechanism of Naringin Combined with Bone Marrow Mesenchymal Stem Cells and Acellular Dermal Matrix on Cartilage Injury. Mod. J. Integrated Traditional Chin. West. Med. 30 (4), 375–380.

[B36] Yuhui Lin X. H. Xiaobin N. Ning H. Qi D. Cheng feng L. Shi ming L. (2011). Transfection of Rat Bone Marrow Mesenchymal Stem Cells with Adenovirus Vector Carrying hBcl -2 and hVEGF165 Genes. Guangdong Med. J. 32 (5). 10.13820/j.cnki.gdyx.2011.05.004

[B37] Zhao X. K. Yu L. Cheng M. L. Che P. Lu Y. Y. Zhang Q. (2017). Focal Adhesion Kinase Regulates Hepatic Stellate Cell Activation and Liver Fibrosis. Sci. Rep. 7 (1), 4032. 10.1038/s41598-017-04317-0 28642549 PMC5481439

[B38] Zou M. Wang F. Gao R. Wu J. Ou Y. Chen X. (2016). Autophagy Inhibition of Hsa-miR-19a-3p/19b-3p by Targeting TGF-β R II during TGF-Β1-Induced Fibrogenesis in Human Cardiac Fibroblasts. Sci. Rep. 6, 24747. 10.1038/srep24747 27098600 PMC4838850

